# A randomised trial of non-invasive cardiac output monitoring to guide haemodynamic optimisation in high risk patients undergoing urgent surgical repair of proximal femoral fractures (ClearNOF trial NCT02382185)

**DOI:** 10.1186/s13741-019-0119-x

**Published:** 2019-08-08

**Authors:** S. J. Davies, D. R. Yates, R. J. T. Wilson, Z. Murphy, A. Gibson, V. Allgar, T. Collyer

**Affiliations:** 1 Department of Anaesthesia, Critical Care and Perioperative Medicine, York Teaching Hospital NHS Foundation Trust, Wiggington Road, York, YO31 8HE UK; 2 Clinical Research Network: Yorkshire and Humber, York Teaching Hospitals Foundation Trust, Wigginton Road, York, UK; 3 Hull York Medical School/Department of Health Sciences, University of York, York, UK; 4 Department of Anaesthesia, Critical Care and Perioperative Medicine, Harrogate and District NHS Foundation Trust, Harrogate, UK

**Keywords:** Hemodynamic, Cardiac Output, Femoral neck fracture

## Abstract

**Background:**

Hip fracture is a procedure with high mortality and complication rates, and there exists a group especially at risk of these outcomes identified by their Nottingham Hip Fracture Score (NHFS). Meta-analysis suggests a possible benefit to this patient group from intravascular volume optimisation. We investigated whether intraoperative fluid and blood pressure optimisation improved complications in this group.

**Methods:**

Patients with a NHFS ≥ 5 were enrolled into this multicentre observer-blinded randomised control trial. Patients were allocated to either standard care or a combination of fluid optimisation and blood pressure control using a non-invasive system. The primary outcome was the number of patients with one or more complications in each group. Secondary outcomes included hospital length of stay (LOS), incidence of hypotension and fluid and vasopressor usage.

**Results:**

Forty-six percent of patients in the intervention group suffered one or more complications compared to the 51% in the control group (OR 0.82 (95% CI 0.49–1.36)). Per-protocol analysis improved the OR to 0.73 (95% CI 0.43–1.24). Median LOS was the same between both groups; however, the mean LOS on a per-protocol analysis was longer in the control group compared to the intervention group (23.2 (18.0) days vs. 18.5 (16.5), *p* = 0.047).

**Conclusions:**

Haemodynamic optimisation including blood pressure management in high-risk patients undergoing repair of a hip fracture did not result in a statistically significant reduction in complications; however, a potential reduction in length of stay was seen.

**Trial registration:**

A randomised trial of non-invasive cardiac output monitoring to guide haemodynamic optimisation in high risk patients undergoing urgent surgical repair of proximal femoral fractures (ClearNOF trial NCT02382185).

## Background

It is estimated that the number of patients that will sustain a hip fracture will reach 100,000 per annum by 2033 with a cost to the UK health services alone of some £ 2.7 billion (White & Griffiths, 2011). Outcomes for this group remain poor with a mortality of 6% at 1 month, increasing to 20–33% (Brauer et al., 2009; Hip Fracture Anaesthesia Sprint Audit Project, n.d.) at 1 year (The National Hip Fracture Database, 2016). Complication rates for this group also remain significant with between 20 and 60% of patients having significant post-operative complications (Roche et al., 2005; Bartha et al., 2013). These complications are not only associated with increased length of stay and healthcare costs, but also reduced long-term survival (Lawrence et al., 2002; Khuri et al., 2005).

Attempts to improve outcomes through fluid optimisation protocols have been mixed (Bartha et al., 2013; Sinclair et al., 1997; Venn et al., 2002) with the most recent work (Moppett et al., 2015) suggesting that fluid optimisation using an arterial waveform-based system did not reduce complications or mortality, although a recent meta-analysis shows a trend towards both. The authors suggested that the role of tighter arterial pressure control should be addressed as part of any future intervention. Given that the incidence of hypotension in the Anaesthesia Sprint Audit of Practice (ASAP) (White et al., 2016) was significant with 56% of patients undergoing neck of femur (NOF) repair having a systolic blood pressure of less than 90 mmHg, and one third having a mean arterial pressure (MAP) of less than 55 mmHg, and the association of hypotension with adverse events (Salmasi et al., 2017; Sun et al., 2015), this may be a valid treatment strategy in this group.

Within this group of elderly patients, a high-risk group exists. The Nottingham Hip Fracture Score (NHFS) is a validated scoring system that predicts patients at increased risk of both 30-day and 1-year mortality (Wiles et al., 2011; Maxwell et al., 2008). This high-risk group, with an increased mortality rate, may also have an increased complication rate, and hence any effective intervention may have a greater impact on these outcomes.

The aim of the study was to investigate the role of haemodynamic optimisation (stroke volume optimisation and blood pressure control) in high-risk patients defined by a NHFS ≥ 5 undergoing urgent surgical repair of proximal femoral fractures and the effects of this on complications. Secondary outcomes included the length of stay, the incidence of hypotension and the use of vasopressors and fluids.

## Methods

We performed a multicentre randomised controlled observer-blinded trial.

This study was approved by the National Research Ethics Service (NRES) committee Yorkshire and the Humber Leeds West (14/YH/1170). Written informed consent was obtained by those individuals that held capacity; otherwise, if a patient met the inclusion criteria but lacked the capacity to consent, we followed the process outlined in the Mental Capacity Act and approved by the NRES committee. The trial was registered prior to patient enrolment at clinicaltrials.gov (NCT02382185).

### Study population

Patients due to undergo urgent or emergency repair of a proximal femoral fracture with NHFS ≥ 5 were enrolled. Patients were excluded if they were aged < 50 years, had an American Society of Anesthesiologists (ASA) physical status classification 5 or had multiple injuries requiring operative management.

### Randomisation, allocation and blinding

Participants were randomised on a 1:1 ratio of ‘conventional fluid therapy’ (control group) or ‘haemodynamic optimisation’ (intervention group). The randomisation was stratified on the basis of the type of anaesthesia (spinal or general anaesthesia) and hospital. Block randomisation was used to ensure a similar number of participants between the intervention and control groups.

The randomisation sequence was prepared using randomisation software, and individual allocations were concealed in sequentially numbered, sealed, opaque envelopes. The unopened envelopes were held securely. The lowest available randomisation numbered envelope was opened by the research nurse once the anaesthetic had been performed.

### Treatment of participants

#### Intervention group

Prior to induction of anaesthesia, an appropriately sized ClearSight™ (Edwards Lifesciences, Irvine, USA) non-invasive haemodynamic monitor probe was placed on a suitable finger and baseline haemodynamic measurements were taken (blood pressure, heart rate, stroke volume, cardiac output). After induction of anaesthesia, the participant’s stroke volume was optimised using 250 ml boluses of Hartmann’s solution. This was defined as repeated boluses of crystalloid until a stroke volume rise of > 10% was no longer seen. The stroke volume (SV) measurement prior to the final fluid bolus was set as the target SV. Once the stroke volume was optimised, mean arterial blood pressure was maintained to within 30% of baseline values using a peripheral phenylephrine infusion or metaraminol infusion. Phenylephrine infusion was at a concentration of 100 μg/ml, whilst metaraminol was at a concentration of 500 μg/ml. Both were started at a rate of 10 ml/h and titrated to response by alteration of the infusion rate in increments of 2 ml/h. After titration of the vasopressor infusion to achieve the desired mean arterial pressure, if the stroke volume increased by greater than 10% from the pre-infusion value, then this was taken as the new baseline value against which further decreases in stroke volume were measured. If the target stroke volume decreased by 10%, a fluid challenge was given, as described previously. If during stroke volume optimisation, prior to SV maximisation, the treating clinician deemed the mean arterial pressure unacceptably low for that patient, they were permitted to administer a bolus of vasopressor at their discretion. Due to the need to not influence the current clinical practice excessively, clinicians were allowed to administer ephedrine if they felt that inotropic support was required. Data on stroke volume, heart rate, blood pressure, cardiac output and oxygen saturation was recorded at baseline and then every 20 s.

#### Control group

Prior to the induction of anaesthesia, a ClearSight™ non-invasive haemodynamic monitor probe was sited and baseline haemodynamic measurements were taken. All fluid management and administration of inotrope or vasopressor therapy were at the discretion of the anaesthetist as per the current practice at the host institution. Only Hartmann’s solution was used as per institutional policies. Data on stroke volume, heart rate, blood pressure, cardiac output and oxygen saturation were recorded at baseline and then every 20 s. The anaesthetist was unable to see the monitor and all alarms were silenced.

The surgeon remained blinded throughout the procedure. Other members of the team attending to participants in the control group were blinded to the readings from the monitor; however, in order to administer the treatment, this was not possible in the intervention group; hence, the anaesthetist was aware of the treatment allocation.

Post-operatively, a research nurse or investigator who was not present in the theatre, and therefore was blinded to group allocation, performed the follow-up visits to assess outcome measures. The post-operative case report form (CRF) was separate from the intraoperative CRF and the two were reconciled at the termination of the study.

### Anaesthetic technique and post-operative care

Anaesthetic technique was at the discretion of the anaesthetist as per the current practice at the host institution in both the treatment and control groups.

Patients were cared for following surgery in the post-anaesthetic care unit (PACU), and post-operative maintenance fluid therapy was administered at a rate of 1 ml/kg/h until oral intake was adequate. The type of crystalloid maintenance fluid was at the discretion of the anaesthetist. Fluid boluses were given if clinically indicated. Vasopressor infusions were reduced to maintain an acceptable mean arterial pressure as determined by the anaesthetist until discontinued. Discharge from the PACU was determined by local protocols.

### Outcome measures

The primary outcome was the number of patients who developed one or more in-hospital post-operative complications as defined by Copeland and modified for this patient group by Bartha (Bartha et al., 2013) (Appendix 1).

Secondary outcomes included the incidence of morbidity at days 3, 5 and 10 as measured by the Post-Operative Morbidity Survey (POMS) (Bennett-Guerrero et al., 1999), length of stay in hospital after surgery, intraoperative haemodynamic variables (stroke volume, heart rate, blood pressure, cardiac output), volume of intraoperative fluid administered, incidence of intraoperative hypotension and the use of intraoperative vasopressor support.

### Statistical analysis

A retrospective analysis of clinical notes identified that 75% of patients undergoing neck of femur repair with a NHFS ≥ 5 had one or more complications as defined in Appendix 1. A meta-analysis by Grocott et al. (Grocott et al., 2013) that reviewed studies designed to increase global blood flow as defined by explicit goals measured either invasively or non-invasively, for example, stroke volume, suggested a RR reduction of 0.68 for complications in patients undergoing major surgery, whilst an economic and feasibility analysis by Bartha et al. (Bartha et al., 2012) suggested the intervention would be cost-effective with a modest effective size (relative risk of 0.84). A clinically significant reduction in the incidence of complications from 60 to 42% during hospital stay would require two groups of patients with 120 patients per group to have an 80% power of detecting a difference with a significance of *p* < 0.05.

Baseline characteristics are compared descriptively in line with CONSORT. Descriptive statistics are presented as mean (SD), minimum-maximum, or median (interquartile range (IQR)) and valid *n* if data is missing. Categorical data is presented as *n* (%) and the valid *n* if data is missing. Both intention-to-treat (ITT) and pre-specified per-protocol (PP) analyses were performed for the primary outcome and for the length of stay.

For the primary outcome, a chi-square test was used to compare the proportion in each group developing one or more in-hospital post-operative complications, and logistic regression was used to calculate the odds ratio.

For the secondary outcomes, chi-square tests were used to compare proportions between groups. *t* tests were used to compare continuous variables or Mann–Whitney tests if data was non-normal or ordinal data. The end of surgery haemodynamic data (stroke volume and cardiac index) was compared between groups using ANCOVA, adjusting for start of surgery values.

For blood pressure measurements for each patient, we calculated the total operation time and for each threshold (MAP 70, 65, 60, 55 and 50), and we calculated the number of minutes below the threshold. The area under the curve (AUC) is therefore the total time under threshold/total operation time. We also calculated the value of MAP as a percentage of baseline (%MAP) and for each threshold calculated the number of minutes below the threshold for %MAP below 10%, 20%, 30%, 40%, 50% and 60% of baseline. The AUC is therefore the total time under threshold/total operation time.

A *p* value of < 0.05 was considered to indicate statistical significance. No adjustments have been made for multiple significance testing. SPSS (V24) was used for statistical analysis.

We updated the Cochrane systematic review using identical search criteria in Cochrane, Medline, EMBASE and trial registries (clinicaltrials.gov and WHO). Data were analysed using the Mantel–Haenszel random effects risk ratios in RevMan V5.3.5.

## Results

**Fig. 1:**
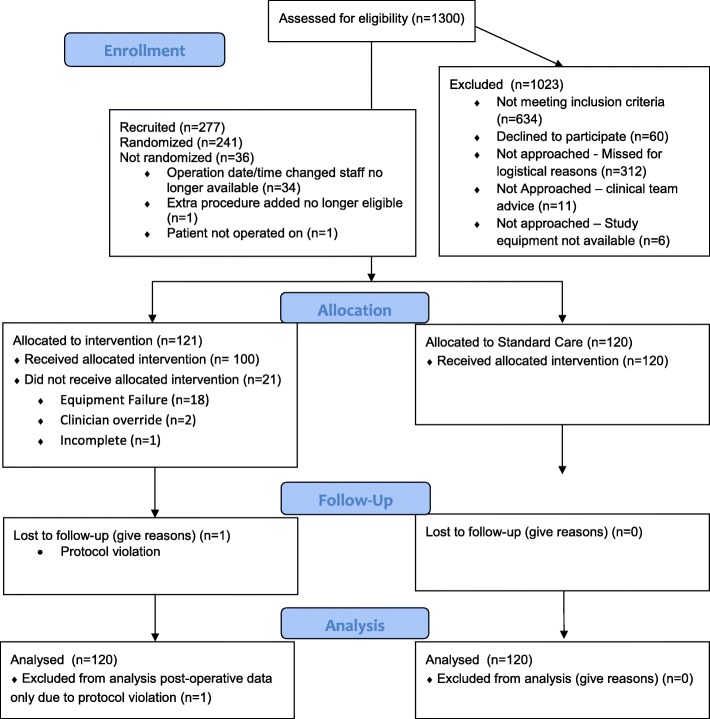
Consort flow diagram

Two hundred forty-one patients were recruited and randomised from November 2015 to September 2017. Consort flow chart for the study is shown in Fig. 1. Patient demographics, surgical and anaesthetic details are shown in Table 1.

**Table 1:** Patient demographics, surgical and anaesthetic details

	Control	Intervention
	*n* = 120	*n* = 121
Age (years)	85.7 (7.5), range 57–103	87.3 (6.1), range 67–98
Gender		
Female	65 (54%)	75 (62%)
Male	55 (46%)	46 (38%)
BMI (kg/m^2^)	23.7 (4.5)	24.0 (4.4)
BSA (m^2^)	1.7 (0.2)	1.7 (0.2)
Co-morbidities		
Hypertension	67 (56%)	63 (53%)
Angina	14 (12%)	12 (10%)
Atrial fibrillation	24 (20%)	34 (28%)
Myocardial infarction	10 (8%)	14 (12%)
CABG	7 (6%)	2 (2%)
Heart failure	7 (6%)	2 (1.7%)
Chronic renal failure	14 (12%)	18 (15%)
Previous stroke/TIA	28 (23%)	16 (13%)
Diabetes	21 (18%)	14 (12%)
COPD	8 (7%)	13 (11%)
Baseline BP		
Systolic (mmHg)	145.0 (27.8), 46–222, *n* = 119	142.5 (25.3), 72–231, *n* = 120
Diastolic (mmHg)	75.1 (14.0), 46–110, *n* = 119	74.1 (14.8), 32–108, *n* = 120
Mean (mmHg)	98.7 (17.6), 61–159	98.9 (17.9), 48–148
ASA grade		
1	0 (0%)	1 (1%)
2	12 (10%)	23 (19%)
3	83 (69%)	75 (62%)
4	25 (21%)	22 (18%)
Anaesthetic		
General	70 (58%)	64 (53%)
Spinal	50 (42%)	56 (47%)
Sedation (% of spinal patients)	45 (90%)	44 (77%)
Block	62 (52%)	64 (53%)
Type of block		
Fascia iliaca	34 (28%)	38 (32%)
Femoral	28 (23%)	24 (20%)
Unknown	0 (0%)	1 (1%)
n/a	58 (48%)	57 (47%)
Grade of surgeon		
Consultant	43 (36%)	42 (35%)
Fellow	0 (0%)	1 (1%)
SPR	55 (46%)	62 (51%)
Staff grade	22 (18%)	14 (12%)
Not known	0 (0%)	1 (1%)
Surgery duration (mins)	69.2 (27.8), 13–181	65.0 (22.4), 6–148
Cemented implant (yes)	58 (48%)	58 (48%)
Implant		
Cannulated screws	3 (3%)	3 (3%)
DHS	37 (31%)	45 (37%)
Gamma nail	13 (11%)	9 (7%)
Hemi-arthroplasty	64 (53%)	59 (49%)
THR	3 (3%)	5 (4%)

Note some percentage in each group may not add up to 100% due to rounding. All numbers are mean (SD) and range unless otherwise expressed as a percentage

### Primary outcome

Complications are shown in Table 2. On the intention-to-treat analysis (ITT), the 55/120 (46%) subjects in the intervention group had a complication compared to the 61/120 (51%) in the control group with an odds ratio of 0.82 (95% CI 0.49–1.36, *p* = 0.439).

**Table 2:** Complications

	Control	Intervention	*p* value
	*n* = 120	*n* = 120	
Cardiovascular	18 (15%)	13 (11%)	0.336
Respiratory	10 (8%)	12 (10%)	0.655
Cerebrovascular	5 (4%)	0 (0%)	0.024
Acute kidney failure	10 (8%)	11 (9%)	0.819
GI bleed	1 (1%)	2 (2%)	0.561
Confusion	19 (16%)	20 (17%)	0.861
Sepsis	8 (7%)	8 (7%)	1.00
DVT	1 (1%)	0 (0%)	0.316
Wound infection	0 (0%)	2 (2%)	0.156
Delayed healing	0 (0%)	2 (2%)	0.156
UTI	10 (8%)	4 (3%)	0.098
Decubitas	1 (1%)	0 (0%)	0.316
Haematoma	1 (1%)	3 (3%)	0.313
Haematological	13 (11%)	12 (10%)	0.883
Number of patients with one or more complications (ITT)	61/120 (51%)	55/120 (46%)	0.439
Number of patients with one or more complications (PP)	61/120 (51%)	43/100 (43%)	0.247

Overall, 21 patients in the intervention group did not receive the intervention; in 18 patients, equipment failure did not allow for the protocol to be delivered, whilst in 2 patients, a clinical decision was made to deviate from the protocol, and in 1 patient, the protocol was not correctly delivered. On a per-protocol analysis (PP), the proportion with at least one complication in the control group was 61/120 (51%) and 43/100 (43%) in the intervention group (OR 0.73 (95% CI 0.43–1.24, *p* = 0.247)).

### Secondary outcomes

#### Length of stay

On ITT analysis, in the control group, the mean LOS was 23.2 (18.0) days (median 16 (11–32)), and in the intervention group, the mean LOS was 18.9 (16.2) days (median 16 (11–23)). There was no statistically significant difference between groups (*p* = 0.053).

On PP analysis, in the control group, the mean LOS was 23.2 (18.0) days (median 16 (11–32)), and in the intervention group, the mean LOS was 18.5 (16.5) days (median 16 (11–23)). There was a statistically significant difference between groups (*p* = 0.047).

### Post-operative morbidity score

There was no difference in POMS score between the groups on any measured day (Appendix 2).

### Mortality

At 30 days, in the control group, 10/120 (8%) had died compared to 11/120 (9%) in the intervention group (*p* = 0.819). At 90 days, in the control group, 27/120 (23%) had died compared to the 25/120 (21%) in the intervention group (*p* = 0.754)

### Fluid volumes and vasopressor doses

Fluid volumes and vasopressor dose are shown in Table 3. The mean total fluid given was 1012.7 ml (354.0) in the control group and 875.0 ml (456.1) in the intervention group (*p* = 0.010). More patients in the control group (47/120) received ephedrine compared to the intervention group (17/103, *p* = 0.000012) and had no statistically significant difference in the total dose. Less patients in the control group received a vasopressor (64/120) compared to the intervention group (115/120, *p* < 0.00001); however, the total dose given was not statistically significant.

**Table 3:** Intraoperative fluid volumes and vasopressor doses (mean (sd), min-max, *n*)

	Control	Intervention	
	*n* = 120	*n* = 120	
Total fluid given	1012.7 (354.0), 200–2000, *n* = 120	875.0 (456.1), 250–2250, *n* = 120	0.010
RBC total	344.2 (116.0), 262–549, *n* = 5	341.8 (129.8), 244–601, *n* = 7	0.976
Platelets	301.0 (0.0) 301–301, *n* = 2	*n* = 0	–
Ephedrine total dose	15.6 (7.9), 6–30, *n* = 47	15.4 (9.8), 6–36, *n* = 17	0.912
Metaraminol total dose	3.2 (2.3), 0.2–8.8, *n* = 48	3.5 (4.5), 0.5–26.5, *n* = 39	0.597
Phenylephrine total dose	2.3 (1.3), 0.4–5.0, *n* = 16	2.2 (1.9), 0.1–12.2, *n* = 76	0.948

### Haemodynamic data

At the start of surgery, the mean stroke volume was 46 (18) ml in the control group and 42 (13) ml in the intervention group (*p* = 0.009). In both groups, the stroke volume had increased at the end of surgery to 48 (17) ml in the control group and 46 (13) ml in the intervention group. There was a significant difference between groups in stroke volume at the end of the surgery, adjusting for start of surgery stroke volume (*p* = 0.028).

At the start of surgery, the mean cardiac index was 2.0 l.min^−1^ m2 (0.7) in the control group and 1.8 l.min^−1^ m^2^ (0.70) ml in the intervention group (*p* = 0.058). In both groups, the cardiac index increased at the end of surgery to 2.1 l.min^−1^ m^2^ (0.7) in the control group and 1.9 l.min^−1^ m^2^ (0.6) in the intervention group. There was no significant difference between groups at the end of surgery, adjusting for start of surgery cardiac index (*p* = 0.320).

The AUC data for the blood pressure data is shown in Table 4. The intervention group spent less time with an absolute MAP below 70, 65 and 60 mmHg. As a percentage of the baseline MAP, the intervention group spent less time below the thresholds of 60%, 50% and 40% of baseline.

**Table 4:** Blood pressure data (AUC)

Blood pressure threshold	Group	Mean (sd)	*p* value
MAP below 70 mmHg	Control	0.37 (0.38)	0.044
	Intervention	0.28 (0.30)	
MAP below 65 mmHg	Control	0.27 (0.31)	0.029
	Intervention	0.18 (0.26)	
MAP below 60 mmHg	Control	0.19 (0.25)	0.043
	Intervention	0.12 (0.20)	
MAP below 55 mmHg	Control	0.11 (0.19)	0.100
	Intervention	0.07 (0.15)	
MAP below 50 mmHg	Control	0.07 (0.13)	0.063
	Intervention	0.03 (0.10)	
MAP below 10 % baseline	Control	0.68 (0.44)	0.652
	Intervention	0.65 (0.38)	
MAP below 20 % baseline	Control	0.53 (0.43)	0.251
	Intervention	0.47 (0.35)	
MAP below 30 % baseline	Control	0.35 (0.36)	0.067
	Intervention	0.27 (0.29)	
MAP below 40 % baseline	Control	0.19 (0.27)	0.036
	Intervention	0.12 (0.19)	
MAP below 50 % baseline	Control	0.08 (0.18)	0.031
	Intervention	0.03 (0.08)	
MAP below 60 % baseline	Control	0.02 (0.07)	0.009
	Intervention	0.00 (0.01)	

There was no association between duration or magnitude of hypotension and complication rates or length of stay.

### Systematic review

We used the same search strategy published in the Cochrane review (Brammar et al., 2013) and the paper by Moppett (Moppett et al., 2015) to update the meta-analysis using identical search criteria in Cochrane, Medline, EMBASE and trial registries (clinicaltrials.gov and WHO). Searches were carried out on 20 November 2018 and repeated on the 6 June 2019. Reports of recent trials and reviews of goal-directed therapy were also hand searched. The methods were based on the PICO format. The eligible population was patients with hip fracture, and the intervention was intraoperative fluid therapy guided by cardiac output monitoring compared with usual care. Additional trials were assessed for the risk of bias using the Cochrane domains. We used a shortened number of outcomes: all-cause mortality within 30 days and the number of survivors with complications. The characteristics and risk of bias were not different to that reported previously (Moppett et al., 2015). We did not find any other ongoing clinical trials or additional trials that had been performed except the one being reported since the updated meta-analysis by Moppett.

Data were analysed using the Mantel–Haenszel random effects risk ratios in RevMan V5.3.

For identical reasons as Moppett, and for comparison, we did not include the trial by Schultz and colleagues (Schultz et al., 1985) and used only the Doppler and control groups, not the CVP-guided group, from Venn and colleagues.

**Fig. 2:**
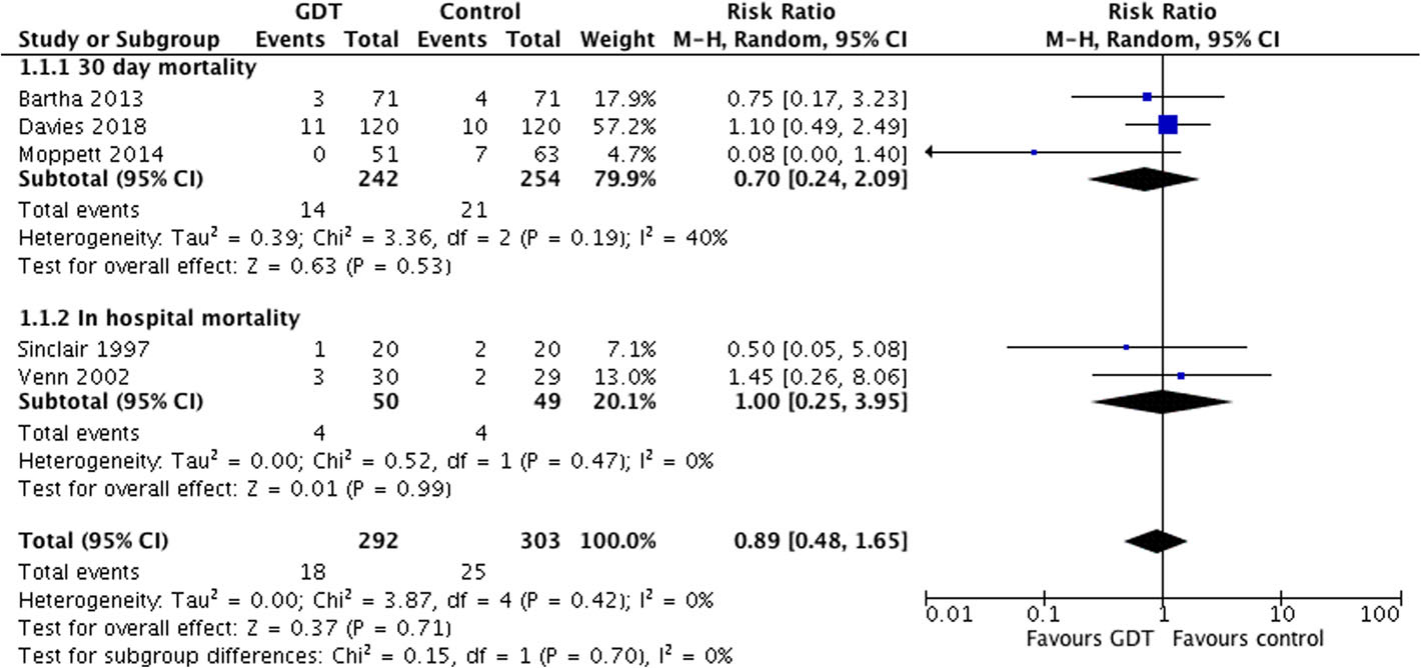
Forest plot of included RCTs of cardiac output-guided fluid therapy during hip fracture surgery assessing mortality (30 days, or in-hospital within 30 days)

**Fig. 3:**
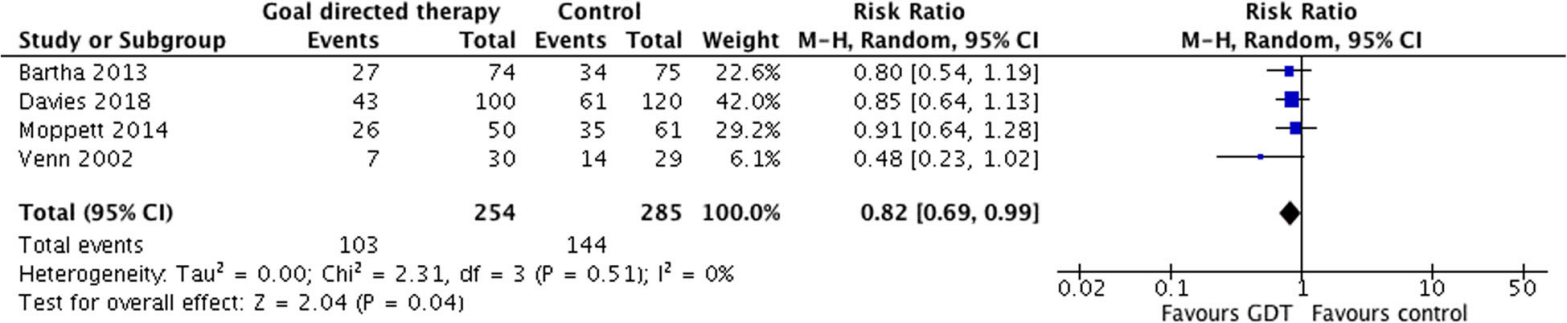
Forest plot of included RCTs of cardiac output-guided fluid therapy during hip fracture surgery assessing post-operative complications

The updated meta-analysis showed no significant effect of the intervention on mortality (Fig. 2) but a significant difference in morbidity: four studies, 539 patients and risk ratio 0.82 (95% CI 0.69–0.99) (Fig. 3).

## Discussion

Haemodynamic optimisation of high-risk patients undergoing proximal neck of femur fracture repair using non-invasive technology did not result in a reduction in post-operative complications or mortality. On the per-protocol analysis, a reduction in mean length of stay was seen.

Whilst the lack of effect is at odds with the original trials using goal-directed fluid therapy in this patient group (Sinclair et al., 1997; Venn et al., 2002), it mirrors the effects seen in the more recent trials where a clinically significant risk reduction was seen that failed to reach significance (Bartha et al., 2013; Moppett et al., 2015).

This trial differs from the more recent ones performed by Bartha and Moppett in various ways; firstly, we used the NHFS to identify those at higher risk of morbidity and mortality. The assumption was that any treatment effect seen from haemodynamic optimisation would be greater in this group. However, it may also be that due to the multiple comorbidities, poor cardiac function and poor outcomes in this group, the outcome is minimally modifiable. Secondly, we chose to enrol patients undergoing both spinal and general anaesthesia, whereas in the work by Moppett and colleagues, only patients undergoing spinal anaesthesia were included, and over 90% of surgery was performed under regional anaesthesia in the work by Bartha. Whilst there is no data that suggests that one form of anaesthesia is related to better outcomes in this patient group (White et al., 2016), it is a potential confounder when comparing the studies. This was stratified for, however, in the randomisation procedure. The inclusion of a population that had a mixture of general and neuraxial anaesthesia is more reflective of the practice seen in the UK and hence makes the results more generalisable. Thirdly, we chose to use a non-invasive measurement of blood pressure and stroke volume in the form of the ClearSight™ system as opposed to an arterial line based system that was used in the 2 recent trials. This approach was chosen as invasive monitoring is rarely used in this patient group and would represent a significant change from practice. The ClearSight™ technology provides the same information from a finger cuff and hence may have greater uptake by the profession as opposed to the increased usage of invasive monitoring. Whilst both systems utilise arterial waveform analysis, the ClearSight™ system reconstructs this from a finger artery waveform and uses pulse contour analysis rather than pulse power analysis as seen in the LiDCO systems. The technology failure rate of 15% in this study is of significance and must be bore in mind by future researchers, with the majority of failure due to poor signal acquisition due to cold peripheries and osteoarthritic fingers. On the per-protocol analysis, a reduction in LOS was seen, confirmed by the meta-analysis, and hence if a suitable technology could be made to work effectively in this group, then there may be a benefit. Finally, and similarly to the work by Bartha, we chose to include a step in the intervention arm that aimed to control for blood pressure. Hypotension has been shown to be associated with acute kidney injury and myocardial injury after non-cardiac surgery (MINS), and a value below 65 mmHg has been suggested as putting individuals at a higher risk of these events (Salmasi et al., 2017; Sun et al., 2015). In addition, hypotension has been associated with harm in this specific patient group (White et al., 2016). We chose to use a treatment value of below 30% from baseline, which has a similar relative risk of injury (Salmasi et al., 2017). Whilst the incidence and duration of hypotension was less in the intervention group, there was no statistical significance in the AUC for MAP below 30% of baseline [0.35 (0.36) vs. 0.27 (0.29), *p* = 0.067] representing a protocol compliance failure for the intervention group overall. Despite this, there was no association between any degree or magnitude of hypotension and outcome in this trial, and the incidence of complications, including kidney injury or cardiovascular complications, was not different between the groups. However, MINS was not specifically screened for and this study was not designed to detect an outcome difference due to variations in MAP. The currently accepted value of 65 mmHg is also based on population data that does not include the group studied in this trial who have a significant incidence of hypertension and in perhaps whom a higher cutoff value relating to outcomes may be seen in this population.

The complication rates in this trial were similar to those seen in the more recent trials. Bartha, who also treated hypotension as part of the protocol, reported a RR of 0.79 (95% CI 0.54–1.16), similar to the OR of 0.73 (95% CI 0.43–1.24) in this trial, whilst Moppett who did not control for baseline blood pressure showed RR of 0.91 (95% CI 0.64–1.28) for patients developing one or more complications. Whilst our groups were well matched in terms of comorbidities, there was a significant difference in stroke volume, with the intervention group having a lower SV at the start of surgery. This may reflect poorer underlying cardiovascular function, and hence this group may have been more compromised compared to the control group leading to a reduced treatment effect. Fluid volumes given were also different between the trials with the intervention group receiving 875 ml of crystalloid compared to 1500 ml in the work by Moppett and colleagues and 1078 ml in the Bartha trial. A concern is that this may relate to an increased use of vasopressor. The use of vasopressors is not without risk, and pure alpha agonists such as phenylephrine are associated with a reduction in cardiac output and cerebral blood flow (Cannesson et al., 2012; Ogoh et al., 2011), depending on preload responsiveness, which may translate into worse outcomes. However, we showed no difference in the amount given between the groups.

A reduction in mean and not median length of stay was detected in patients treated on a per-protocol basis. Whilst this analysis was defined a priori, caution must be used in the interpretation of this result as the trial was not powered to detect this and the effect size is borderline. The mean length of stay is more reflective of the economic impact that an intervention may have on a population, and indeed if this effect can be repeated then due to the magnitude of reduction seen, this would have a significant impact for the NHS. In addition, there was a noticeable reduction in the upper quartile limit for the median stay in the intervention group from 32 to 22 days in keeping with a mean reduction. It may well be that these metrics represent a reduction in the variation of care, which in itself has been suggested to lead to improved outcomes.

An updated meta-analysis showed a significant effect on morbidity with a risk ratio of 0.81 (0.67–0.98) whilst the effects on mortality remained non-significant and unchanged. It is interesting to note that none of the 4 studies included have suggested harm from the intervention; however, given the potential impact that even a small reduction in either LOS or complications would have on such a large population, and the signal seen in meta-analysis, then a large pragmatically designed multicentre trial that addresses both fluid management and blood pressure control is now warranted.

The study has limitations: primarily in that it is underpowered. We expected a complication rate of 60% in the control group and this was only 51%. This may reflect temporal improvements in perioperative care based on guidelines from groups such as the National Institute for Healthcare and Excellence, the National Hip Fracture Database and the Association of Anaesthetists of Great Britain and Ireland amongst others, and additionally, the trial was conducted over the best part of 2 years and there is a chance of a significant Hawthorne effect. Despite this, it remains the largest randomised controlled trial of haemodynamic optimisation of hip fracture patients and adds to the literature and updated meta-analysis. We chose to use the same basket of complications as those used in the Bartha trial in order that direct comparison may be made; however, it may be that these complications are too generic and more procedure specific and patient-relevant end points are needed. Finally, protocol compliance may have been suboptimal as there was no significant difference in patients with a MAP of less than 30% of baseline and a relatively small increase in SV. This may reflect the relatively short nature of the surgery and hence a limited opportunity of optimising patients.

The strengths of the study include a homogenous population, the inclusion of MAP control in the algorithm, the exclusion of lower risk patients and the fact that the intervention was performed in the context of best practice for fractured neck of femur care.

The updated meta-analysis suggests a possible reduction in complications, and the relative reduction in complications appears consistent throughout the three more recent trials. Given the significant and growing volume of surgery undertaken, and the economic impact on health care, larger studies should address a treatment strategy where both fluid and blood pressure management are addressed.

## Data Availability

The datasets used and/or analysed during the current study are available from the corresponding author on reasonable request.

## Appendix 1

**Table 5:** Definitions of post-operative complications

Complication	Definition
Cardiovascular	Acute atrial fibrillation and/or troponin-I (> 50 ng∙l^−1^) and/or angina and/or ECG-detected ischaemia and/or heart failure (X-ray or clinical symptoms requiring changes in the post-operative therapy) and/or CT verified pulmonary embolisation
Respiratory	Need of oxygen > 4 L/min (to maintain SpO_2_> 92%) and CRP > 100 and/or pneumonia diagnosed by X-ray and treated by antibiotics
Cerebrovascular	Focal symptoms and CT scan-verified acute pathology of a stroke or a history and examination consistent with a transient ischaemic attack
Acute kidney failure	30% increase of baseline creatinine or/and < 0.5 ml/h diuresis
Gastrointestinal bleeding	Verified by loss of haemoglobin (Hb < 100 g/l) and blood in the faeces
Confusion	AMTS or answering the question “Do you think the patient had been more confused lately?”
Sepsis	Body temperature < 36° or > 38° and heart rate > 90/min and respiratory rate > 20/min or pCO_2_< 4.3 kPa and leukocytes < 4000 cells/mm^3^ or > 12 000 cells/mm^3^
Deep-vein thrombosis	When suspected and detected by Doppler ultrasonography
Wound infection	Deep and/or superficial with purulent exudate and treated by antibiotics
Delayed healing	Superficial or deep wound breakdown needing surgical intervention
Urinary tract infection	Positive urine culture and/or new clinical symptoms and CRP > 100
Decubitus	Broken skin, but intact subcutaneous tissue or sore involving deep soft subcutaneous tissue without muscle or tissue loss, including soft tissues
Wound haematoma	Needing surgical drainage
Haematological	Requirement in the last 24 h of blood products

## Appendix 2

**Table 6:** POMS scores on post-operative days 3, 5 and 10

		Group				*p* value
		Control		Intervention		
		Count	Column, *N* (%)	Count	Column, *N* (%)	
POMS, day 3 score	0	42	35.0	41	34.2	0.617
	1	46	38.3	52	43.3	
	2	19	15.8	20	16.7	
	3	11	9.2	5	4.2	
	4	2	1.7	2	1.7	
	Total	120	100.0	120	100.0	
POMS, day 3	0	42	35.0	41	34.2	0.892
	>0	78	65.0	79	65.8	
	Total	120	100.0	120	100.0	
POMS, day 5 score	0	56	48.7	57	52.8	0.222
	1	37	32.2	40	37.0	
	2	13	11.3	7	6.5	
	3	9	7.8	3	2.8	
	5	0	0.0	1	0.9	
	Total	115	100.0	108	100.0	
POMS, day 5	0	56	48.7	57	52.8	0.542
	>0	59	51.3	51	47.2	
	Total	115	100.0	108	100.0	
POMS, day 10 score	0	55	60.4	52	57.1	0.634
	1	25	27.5	30	33.0	
	2	8	8.8	5	5.5	
	3	2	2.2	3	3.3	
	4	1	1.1	0	0.0	
	5	0	0.0	1	1.1	
	Total	91	100.0	91	100.0	
POMS, day 10	0	55	60.4	52	57.1	0.651
	>0	36	39.6	39	42.9	
	Total	91	100.0	91	100.0	
